# Platelet-Based Biomarkers for Diagnosis and Prognosis in COVID-19 Patients

**DOI:** 10.3390/life11101005

**Published:** 2021-09-24

**Authors:** Ricardo Wesley Alberca, Rosa Liliana Solis-Castro, Maria Edith Solis-Castro, Fernanda Cardoso, Alberto Jose da Silva Duarte, Luana de Mendonça Oliveira, Nátalli Zanete Pereira, Sarah Cristina Gozzi-Silva, Emily Araujo de Oliveira, Valeria Aoki, Raquel Leao Orfali, Danielle Rosa Beserra, Milena Mary de Souza Andrade, Maria Notomi Sato

**Affiliations:** 1 Laboratory of Dermatology and Immunodeficiencies, LIM-56, Department of Dermatology, School of Medicine and Institute of Tropical Medicine of São Paulo, University of São Paulo, São Paulo 01246-903, Brazil; cardosofer1989@gmail.com (F.C.); adjsduar@usp.br (A.J.d.S.D.); luana.mendonca@usp.br (L.d.M.O.); natalli@usp.br (N.Z.P.); sarahgozzi@usp.br (S.C.G.-S.); emily.araujooliveira@gmail.com (E.A.d.O.); valeria.aoki@hc.fm.usp.br (V.A.); raquelleao@usp.br (R.L.O.); daniellerb@usp.br (D.R.B.); milenaandrade@usp.br (M.M.d.S.A.); marisato@usp.br (M.N.S.); 2 Departamento Académico de Biología y Bioquímica, Facultad de Ciencias de la Salud, Universidad Nacional de Tumbes, Av. Universitaria s/n, Tumbes 24000, Peru; rsolisc@untumbes.edu.pe; 3 Departamento Académico de Medicina Humana, Facultad de Ciencias de la Salud, Universidad Nacional de Tumbes, Av. Universitaria s/n, Tumbes 24000, Peru; esolisc@untumbes.edu.pe; 4 Institute of Biomedical Sciences, University of São Paulo, São Paulo 05508-000, Brazil

**Keywords:** COVID-19, SARS-CoV-2, platelet, coagulation, thrombocytopenia, inflammation, biomarker, prognosis

## Abstract

Coronavirus disease 2019 (COVID-19) caused millions of deaths worldwide. COVID-19’s clinical manifestations range from no symptoms to a severe acute respiratory syndrome, which can result in multiple organ failure, sepsis, and death. Severe COVID-19 patients develop pulmonary and extrapulmonary infections, with a hypercoagulable state. Several inflammatory or coagulatory biomarkers are currently used with predictive values for COVID-19 severity and prognosis. In this manuscript, we investigate if a combination of coagulatory and inflammatory biomarkers could provide a better biomarker with predictive value for COVID-19 patients, being able to distinguish between patients that would develop a moderate or severe COVID-19 and predict the disease outcome. We investigated 306 patients with COVID-19, confirmed by severe acute respiratory syndrome coronavirus 2 RNA detected in the nasopharyngeal swab, and retrospectively analyzed the laboratory data from the first day of hospitalization. In our cohort, biomarkers such as neutrophil count and neutrophil-to-lymphocyte ratio from the day of hospitalization could predict if the patient would need to be transferred to the intensive care unit but failed to identify the patients´ outcomes. The ratio between platelets and inflammatory markers such as creatinine, C-reactive protein, and urea levels is associated with patient outcomes. Finally, the platelet/neutrophil-to-lymphocyte ratio on the first day of hospitalization can be used with predictive value as a novel severity and lethality biomarker in COVID-19. These new biomarkers with predictive value could be used routinely to stratify the risk in COVID-19 patients since the first day of hospitalization.

## 1. Introduction

The coronavirus disease 2019 (COVID-19), caused by the severe acute respiratory syndrome coronavirus 2 (SARS-CoV-2), caused millions of deaths worldwide. COVID-19 severity degree ranges from asymptomatic to a severe systemic disease with respiratory and/or multiorgan damage, and a clinical course that can rapidly progress to deadly complications [1]. Comorbidities such as respiratory disorders [2], organ transplant recipients [3], metabolic disorders [4], and old age [5] are frequently associated with a worse clinical outcome in SARS-CoV-2-infected patients.

Several studies have reported increasing biomarkers in COVID-19 patients, which further increases according to the severity of the disease [5,6,7]. Clinical features and laboratory parameters such as neutrophil-to-lymphocyte ratio (NTL), C-reactive protein (CRP), creatinine, urea, and lactate dehydrogenase (LDH) [5,8,9,10] are used as indicators of COVID-19 severity.

During SARS-CoV-2 infection, aberrant cytokine production referred to as the cytokine storm can occur [11]. The cytokine storm is characterized by an increase in blood levels of interferon-inducible protein 10 (IP10), monocyte chemoattractant protein (MCP-1), macrophage inflammatory protein (MIP)1A and MIP1B, platelet derived growth factor (PDGF), tumor necrosis factor (TNF), interleukin (IL)-1, IL-7, IL-8, IL-9, and interferon (IFN)-γ [5], which contribute to hyper inflammation, increasing the recruitment and activation of immune cells and tissue injury [12,13].

D-dimer and platelet count have also recently been proposed as COVID-19 severity-associated biomarkers due to the central role of coagulation disorders and platelets in coronaviruses immunopathology [14]. COVID-19 increases the circulating levels of platelet factor 4, soluble P-selectin, and thrombopoietin, inducing hyperactivation in the platelets [15]. In addition, several factors in the plasma that can induce hypercoagulation, neutrophils activation, and neutrophil extracellular traps (NETs) production also corroborate platelet activation and the establishment of immunothrombosis [12].

Comorbidities associated with increased risk or severe COVID-19, such as obesity, systemic arterial hypertension, diabetes mellitus, co-infections, and other comorbidities [16,17,18,19], and also immobilization, dehydration, and assisted mechanical ventilation could contribute to coagulatory disorders [20].

Due to the complex interactions between coagulatory and pro-inflammatory factors during COVID-19, we investigate if the conjunction of these factors could be a better biomarker for COVID-19 severity and prognosis. Therefore, we retrospectively analyzed all biomarkers available on the first day of hospitalization, before any treatment, to investigate if a combination of coagulatory factors and inflammatory biomarkers could be used as a diagnostic and prognostic biomarker in patients with COVID-19. 

## 2. Materials and Methods

We recruited 367 patients at the Hospital das Clínicas of the University of São Paulo (HCFMUSP), a public tertiary hospital with a general ward (GW) and intensive care unit (ICU) for COVID-19 patients. Inclusion criteria were as follows: diagnosed with SARS-CoV-2 infection by the detection of SARS-CoV-2 RNA (E gene and N gene, with endogenous control with RNAseP), by reverse-transcriptase polymerase chain reaction (RT-PCR) in a nasal swab, with a detection limit of 40 copies of viral RNA/reaction [21]. The equipment used for RT-PCR was the Abbott m2000sp nucleic acid extractors and the thermal cycler, Abbott m2000rt from Abbott Laboratories Inc. All laboratory data analysis were performed with Cobas 8100 Automated Workflow Series with a post-analytical unit (Roche Diagnostics, Basel, Switzerland).

**Figure 1:**
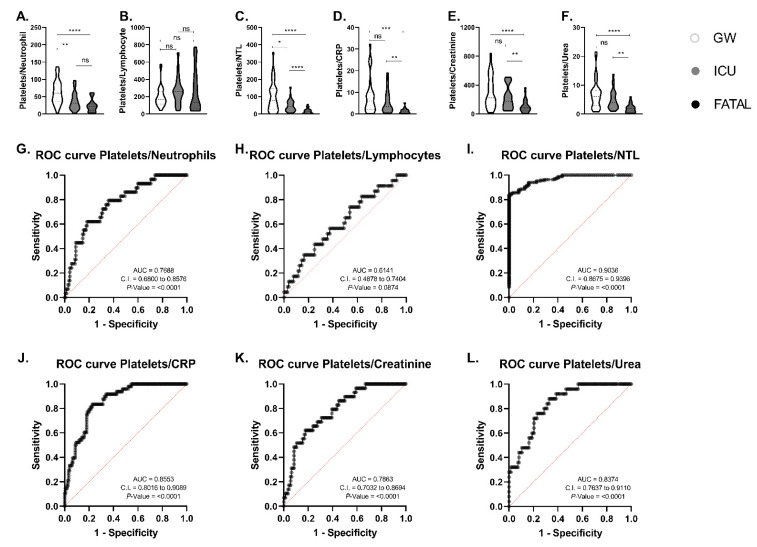
Clinical features of COVID-19 patients on the day of hospitalization. (**A**) Platelets/neutrophils, (**B**) platelets/lymphocytes, (**C**) platelets/NTL, (**D**) platelets/CRP, (**E**) platelets/creatinine, and (**F**) platelets/urea ratios on the first day of hospitalization. Receiver operating characteristics and area under the curve with multiple logistic regression test on (**G**) platelets/neutrophils, (**H**) platelets/lymphocytes, (**I**) platelets/NTL, (**J**) platelets/CRP, (**K**) platelets/creatinine, and (**L**) platelets/urea ratios on the first day of hospitalization. GW: COVID-19 patients with moderate COVID-19 in the general ward; ICU: COVID-19 patients with severe COVID-19 in the intensive care unit; FATAL: COVID-19 patients that died due to COVID-19. Non-statistically significant (NS) * <0.05, ** <0.01, *** <0.001, and **** <0.0001 difference between groups. Kruskal–Wallis test with Dunn’s multiple comparisons. AUC = area under the curve, C.I. = confidence interval. Data was collected between 1 August 2020 and 30 December 2020.

Patients without comorbidities or with type 1 or type 2 diabetes mellitus, obesity, systemic arterial hypertension, heart- (cardiopathy, atrial tachycardia, cardiac insufficiency, heart transplant recipient), kidney- (chronic kidney disease or kidney transplant recipient), or hepatic- (liver transplant recipient) associated comorbidities were included. Exclusion criteria were the presence of neoplasias, immunodeficiencies, or co-infections (bacterial or viral). A total of 306 patients was included in the investigation. A total of 231 patients survived COVID-19, namely 169 patients in the GW and 62 patients in the ICU. Admission to the ICU was solely based on patients’ illness and the necessity for invasive mechanical ventilation. The hospital was not in a shortfall of ICU units. FATAL groups consisted of 75 patients that were initially hospitalized in the GW (9 patients) and ICU (66 patients) that died due to COVID-19. The 9 patients from the FATAL group that were initially hospitalized in the GW were transferred to the ICU as the disease progressed. This investigation was approved by the Ethics Committee of Hospital das Clínicas da Faculdade de Medicina da Universidade de São Paulo—HCFMUSP (no. 30800520.7.0000.0068-2020) and performed in conformity with the 2013 revision of the Declaration of Helsinki. The SARS-CoV-2 detection test, EDTA blood sample collection, and analysis were performed on the first 24 h after hospitalization. Table data are shown as mean and standard error of the mean (SEM), and Figure 1 data are shown in violin plots or area under the curve (AUC) and receiver operating characteristics (ROC) with confidence interval (C.I.). Statistical analysis was performed with the Kruskal–Wallis test for multiple comparisons and multiple logistic regression tests on all proposed biomarkers, with controlled variables, namely outcome, age, sex, and the biomarker. The Pearson correlation test was used for outcomes and the platelets/NTL ratio with GraphPad Prism 9 software (GraphPad Software, Inc., San Diego, CA, USA). 

## 3. Results

Patients from GW, ICU, and FATAL did not present any age differences (Table 1). Laboratory data identified an increase in D-dimer levels in the FATAL group in comparison to GW, but not ICU (Table 1). Blood glucose was increased in the FATAL group in comparison to ICU, but not GW. Additionally, lactate dehydrogenase was increased in ICU and FATAL groups in relation to GW, but no difference between ICU and FATAL was verified (Table 1).

We verified an increase in the number of neutrophils and NTL in the ICU and FATAL groups in comparison with GW, but no difference among ICU and FATAL groups (Table 1). Lymphocytes count, CRP, creatinine, urea, and platelets were increased in the FATAL group in relation to the GW group, but not in comparison to the ICU group (Table 1). Since these biomarkers failed to identify differences between all groups, we investigated if the ratio between platelets and other markers could present a better sensitivity to the disease outcome. We verified statistically significant differences in the platelets/neutrophils, platelets/CRP, platelets/creatinine, and platelets/urea ratios between GW and FATAL and between ICU and FATAL groups (Figure 1A,D–F). 

No difference was verified between groups in the platelets/lymphocyte ratio (Figure 1B). Finally, using the formulation platelets count/(neutrophils-to-lymphocyte ratio), we generated a platelets/NTL that was sensitive enough to verify differences between all groups (Figure 1C), both in the severity—distinguishing if the patient was going to need or not intensive care assistance and assisted mechanical ventilation—and also the outcome of COVID-19 patients, namely survival and non-survival.

We perform a multiple logistic regression test on all proposed biomarkers in all patients, with controlled variables including the outcome (survived and non-survived), age, sex, and the biomarker. We verified that the ratio between platelets/neutrophils, platelets/NTL, platelets/CRP, platelets/creatinine, and platelets/urea provided a statistically significant biomarker for the prediction of death due to COVID-19 (Figure 1G,I–L). As expected, the ratio between platelets and lymphocytes was not associated with COVID-19 outcomes (Figure 1H). From all the proposed biomarkers, the platelets/NTL ratio provided the greater area under the curve (AUC) of 0.9036 (Figure 1I). The platelets/NTL ratio and the outcome also resulted in a moderate correlation by the Pearson correlation test, with a 0.54 size of correlation [22].

## 4. Discussion

Previous reports have designed models for predicting COVID-19 severity and outcomes based on the laboratory data of the first day after hospitalization [5,8,9]. In opposition, previous reports have failed to observe differences in commonly used inflammatory biomarkers on the day of hospitalization and favored longitudinal investigation [2,23,24]. Due to the complexity of COVID-19, it is expected that a single biomarker would not be able to predict the patients´ outcomes. Therefore, we investigated and proposed a novel biomarker that would conciliate both the inflammation and hypercoagulation observed in SARS-CoV-2 infected patients, and that could both predict COVID-19 severity and risk of death on the first day of hospitalization.

Recognized biomarkers in COVID-19 and other respiratory infections such as neutrophil count, C-reactive protein, D-dimer, creatinine, and urea in the blood, can distinguish SARS-CoV-2 infected from non-infected patients and are further increased in more severe patients [9,25,26]. Although previous manuscripts identified an increase in those biomarkers, no association with patient outcomes was performed. 

A previous analysis identified that lymphopenia, elevated CRP, and elevated creatinine are associated with organ injury [27] and a higher risk of death [28]. During COVID-19, patients commonly present elevated counts of leukocytes, mainly neutrophils, associated with lymphopenia, in a process of immune dysregulation [29]. In our cohort, we identified an increase in creatinine, urea, CRP, and D-dimer levels as well as lymphopenia only in the FATAL group in relation to the GW group, but not in relation to ICU patients. 

Platelets can interact with innate immune cells such as neutrophils and macrophages [30] and the production of cytokines and chemokines [31] and interact with injured endothelia [31]. Recently, platelet count has been proposed as a severity biomarker in COVID-19 [32], and thrombocytopenia at the first day of hospitalization has been proposed as a biomarker for a higher mortality rate [33]. To our surprise, in our cohort, thrombocytopenia could not be used as a severity biomarker since it failed to distinguish between patients in the GW and ICU.

Hypercoagulability has also been identified in other coronavirus infections, including severe acute respiratory syndrome 1 (SARS-CoV-1) and the Middle East respiratory syndrome (MERS-CoV) [14]. The hypercoagulability caused by the SARS-CoV-2 may be generated or increased by the pro-inflammatory response and endothelial damage [34], which also occurs in dengue infection [35]. In addition, COVID-19’s main causes of death are often related to respiratory failure, venous thromboembolism, or pulmonary embolism [36,37]. Due to the central role of both inflammation and coagulatory disorders, we investigated if a platelet-based biomarker, with data from the first day of hospitalization, could distinguish patients according to COVID-19 severity and predict the disease outcome.

We identified that the ratio between platelets and inflammatory biomarkers, such as platelets/neutrophils, platelets/NTL, platelets/CRP, platelets/creatinine, or platelets/urea levels from the first day of hospitalization, were associated with patient outcomes. All these biomarkers were reduced in ICU patients and further reduced in patients that died due to COVID-19. These data support previous investigations about the central role of platelets in COVID-19 [30,32,33].

To evaluate the predictive value of these biomarkers, we performed a multiple logistic regression test with controlled variables [38]. We identified that our new proposed biomarker ratios—platelets/neutrophils, platelets/CRP, platelets/creatinine, platelets/urea, and platelets/NTL—could be used to predict patient outcomes. These results present an alternative that is easy to measure and calculate in clinical practice compared to previously reported predictive biomarkers [39,40]. A similar biomarker called the systemic immune-inflammation index (SII) also consider neutrophil, platelet, and lymphocyte counts [41]. In SII, the number of neutrophils is multiplied by the platelet counts and divided by the number of lymphocytes [41]. SII is commonly used in patients with high platelet counts, such as in patients with solid tumors [42]. Since COVID-19 causes a reduction in the platelet count, we hypothesize that our biomarker is better suited for COVID-19 and other infections with a high neutrophil and platelet count.

Our work demonstrates the importance of the immune activation and coagulatory disorders in COVID-19 and strengthens the possible use of therapies to control both inflammatory disorders [43] and also platelet-based treatments such as the use of Bruton tyrosine kinase (Btk) to curb platelet activation, immunothrombosis, and the formation of platelet–neutrophil aggregates [44].

It is important to highlight that our work presents limitations such as the distribution of comorbidities between groups, and the low presence of patients without comorbidities. In addition, these biomarkers should be tested with other SARS-CoV-2 variants and in larger cohorts.

## 5. Conclusions

In summary, we demonstrated that several biomarkers are increased at the first day of hospitalization; nevertheless, they fail to identify the disease outcome. We proposed a few novel biomarkers that encompass the proinflammatory and hypercoagulation states during COVID-19 in the first day of hospitalization, to more precisely determine the severity and outcomes of patients.

## Figures and Tables

**Table 1:** Patients’ characteristics by the severity of the disease.

Male/Female	91/78		40/22		48/27			
Laboratory Data	MEAN	SEM	MEAN	SEM	MEAN	SEM	Reference Numbers	*p*-Value
**Age (years)**	56.24	0.98	56.93	1.3	60.19	1.39		0.0736
**Neutrophils (×10^3^/mm^3^)**	**5.805**	**0.5578**	**9.621**	**1.172**	11.11 *^,#^	**1.165**	**2.5–7.5**	**0.0002**
**Lymphocytes (×10^3^/mm^3^)**	**1.438**	**0.1312**	**1.329**	**0.1266**	1.019 *	**0.1609**	**1.5–3.5**	**0.0266**
**Neutrophil-to-lymphocyte ratio (NTL)**	**4.566**	**0.3937**	**8.432**	**0.8036**	11.55 *^,#^	**1.159**	**4–11**	**<0.001**
**Creatinine (mg/dL)**	**1.68**	**0.2789**	**2.085**	**0.3353**	2.851 *	**0.4301**	**0.7–1.2**	**0.0197**
**Urea (mg/dL)**	**58.02**	**6.372**	**80.68**	**11.25**	116.7 *	**13.45**	**10–50**	**0.0003**
**C-reactive protein (CRP) (mg/L)**	**68.18**	**15.16**	**104.9**	**21.15**	146 *	**26.42**	**<5.0**	**0.0490**
**Platelets (×10^3^/mm^3^)**	**296.7**	**15.79**	**297.1**	**21.99**	229.9 *	**22.58**	**150–400**	**0.0131**
**Alanine aminotransferase (U/L)**	36.59	5.593	68.46	13.52	39.47	6.877	<41	0.1206
**Aspartate aminotransferase (U/L)**	37.88	6.964	44.23	7.943	49.07	6.916	<37	0.3123
**Glutamyl transferase gamma (U/L)**	115.3	43.86	334.8	164.1	295.0	77.64	8–61	0.0689
**Glucose (mg/dL)**	**160.8**	**45.49**	**172.1 ^#^**	**39.99**	**288.2 ^#^**	**32.03**	**70–100**	**0.0073**
**Lactate dehydrogenase (U/L)**	**280**	**26.41**	**505.2**	**53.78**	515 *^,#^	**48.03**	**135–225**	**0.0038**
**D-dimer (ng/mL)**	**1443**	**281.5**	**6590**	**2642**	6191 *	**2461**	**<500**	**0.0237**
Comorbidities								
**Diabetes mellitus/** **Metabolic syndrome**	76		46		27			
**Systemic Arterial Hypertension**	77		49		35			
**Heart disease**	15		10		6			
**Hepatic disease**	10		4		3			
**Renal disease**	19		7		12			
**No disease**	7		3		4			

* *p* < 0.05 difference from LETHAL to GW group, ^#^ *p* < 0.01 difference from LETHAL to ICU group. Bold: indicates biomarkers with differences between groups. Reference values from Divisão de Laboratorio Central do HC/FMUSP.

## Data Availability

The data presented in this study are available upon request from the corresponding author.
